# AI reshaping life sciences: intelligent transformation, application challenges, and future convergence in neuroscience, biology, and medicine

**DOI:** 10.3389/fdgth.2025.1666415

**Published:** 2025-09-23

**Authors:** Jiahuan Gong, Zihao Zhao, Xinxin Niu, Yanan Ji, Hualin Sun, Yuntian Shen, Bingqian Chen, Bei Wu

**Affiliations:** ^1^ Key Laboratory of Neuroregeneration of Jiangsu and Ministry of Education, Medical School of Nantong University, Co-Innovation Center of Neuroregeneration, Nantong University, Nantong, China; ^2^ Department of Orthopedics, First People’s Hospital of Changshu City, Changshu, China; ^3^ Research Office in Ideology and Political Education, School of Marxism, Nantong University, Nantong, China

**Keywords:** artificial intelligence, neuroscience, biology, medicine, application challenge

## Abstract

The rapid advancement of artificial intelligence (AI) is profoundly transforming research paradigms and clinical practices across neuroscience, biology, and medicine with unprecedented depth and breadth. Leveraging its robust data-processing capabilities, precise pattern recognition techniques, and efficient real-time decision support, AI has catalyzed a paradigm shift toward intelligent, precision-oriented approaches in scientific research and healthcare. This review comprehensively reviews core AI applications within these domains. Within neuroscience, AI advances encompass brain-computer interface (BCI) development/optimization, intelligent analysis of neuroimaging data (e.g., fMRI, EEG), and early prediction/precise diagnosis of neurological disorders. In biological research, AI applications include enhanced gene-editing efficiency (e.g., CRISPR) with off-target effect prediction, genomic big-data interpretation, drug discovery/design (e.g., virtual screening), high-accuracy protein structure prediction (exemplified by AlphaFold), biodiversity monitoring, and ecological conservation strategy optimization. For medical research, AI empowers auxiliary medical image diagnosis (e.g., CT, MRI), pathological analysis, personalized treatment planning, health risk prediction with lifespan health management, and robot-assisted minimally invasive surgery (e.g., da Vinci Surgical System). This review not only synthesizes AI's pivotal role in enhancing research efficiency and overcoming limitations of conventional methodologies, but also critically examines persistent challenges, including data access barriers, algorithmic non-transparency, ethical governance gaps, and talent shortages. Building upon this analysis, we propose a tripartite framework (“Technology-Ethics-Talent”) to advance intelligent transformation in scientific and medical domains. Through coordinated implementation, AI will catalyze a transition toward efficient, accessible, and sustainable healthcare, ultimately establishing a life-cycle preservation paradigm encompassing curative gene editing, proactive health management, and ecologically intelligent governance.

## 1 Introduction

Artificial intelligence (AI), among the most revolutionary technologies of the 21st century, is fundamentally restructuring scientific research paradigms (shifting from experience-driven to data-algorithm symbiosis), healthcare delivery architectures (rebuilding the prevention-diagnosis-treatment continuum), and pedagogical methodologies (enabling transition from standardized instruction to personalized cognitive mapping) (1, 2). The core drivers of AI's reconstruction of three knowledge-production systems stem from intelligent deconstruction of massive heterogeneous data (overcoming traditional processing bottlenecks), deep pattern recognition in complex biological systems (revealing multidimensional correlations), and real-time responsiveness in dynamic environments (enabling millisecond-resolution decisions) (3). Within the life sciences nexus—spanning neuroscience, biology, and medicine—these capabilities generate cascading effects that systematically transition disciplinary paradigms toward intelligence-augmented frameworks.

Neuroscience grapples with the complexity of dynamic neural circuitry analysis and multimodal signal integration; biology urgently requires processing exponentially growing genomic-to-proteomic data deluges; while clinical medicine demands precision decision-making frameworks spanning the disease prevention-diagnosis-treatment continuum (4–7). A core challenge shared by these three fields lies in extracting actionable knowledge from an expanding data universe (8, 9). Whereas conventional approaches are constrained by computational inefficiency and analytical dimensionality, AI technologies—leveraging machine learning, deep learning, and related algorithms—overcome these barriers to enable profound mining of complex biological principles. This breakthrough provides transformative solutions for scientific discovery and clinical interventions.

Notably, AI's interdisciplinary nature intrinsically catalyzes convergent innovation across domains (10, 11). This convergence transcends technological integration to drive methodological reconceptualization. The personalized learning frameworks from intelligent education synergistically converge with precision medicine's individualized intervention logic, collectively establishing data-driven service paradigms. Simultaneously, real-time feedback mechanisms for learning assessment share core decision architectures with dynamic monitoring technologies in medical imaging analytics (12, 13). The closed-loop “analysis-internalization-remediation” cycle in education exhibits methodological resonance with medicine's prevention-diagnosis-treatment continuum (14). These cross-domain synergies position AI as a pivotal nexus connecting neuroscience, biology, and medicine, fostering an emergent integrative research ecosystem.

This review examines the AI-driven intelligent transformation surge across the life sciences. Through systematic analysis of signature applications in neuroscience [brain-computer interfaces (BCI), neuroimaging analytics], biology (gene-editing optimization, protein structure prediction), and medicine (intelligent diagnostics, surgical robotics), we elucidate how AI technologies reconfigure research trajectories and clinical practices. Concurrently, we critically dissect core challenges in data governance, algorithmic trustworthiness, and ethical regulation, ultimately projecting innovation pathways for cross-disciplinary convergence that catalyze paradigm shifts in life sciences. This narrative review synthesizes recent advances in AI applications across neuroscience, biology, and medicine, drawing on seminal and emerging literature to highlight transformative trends and challenges.

To systematically navigate these transformative opportunities and inherent challenges, this review proposes and adopts a tripartite “Technology-Ethics-Talent” framework. This integrative lens serves as the foundational structure for our analysis, positing that the sustainable and equitable advancement of AI in the life sciences necessitates simultaneous progress in three core dimensions: technological innovation (e.g., developing robust, interpretable algorithms and secure data infrastructures), ethical governance (e.g., establishing accountable, transparent, and fair regulatory protocols), and talent cultivation (e.g., fostering interdisciplinary experts fluent in both computational and domain-specific knowledge). This framework not only organizes our critical examination of current applications and challenges but also underpins our forward-looking recommendations for achieving a convergent and responsible intelligent transformation across neuroscience, biology, and medicine.

This narrative review was based on a broad literature search in PubMed, IEEE Xplore, and arXiv using keywords including “AI in neuroscience”, “AI in drug discovery”, “AI in medical imaging”, “ethical AI”, etc., focusing on high-impact publications from 2020 to 2024.

## 2 AI revolutionizes neuroscience: three paradigm-shifting applications

The core challenge in neuroscience lies in deciphering highly complex brain functional networks and neurological disease mechanisms. AI, leveraging its capacity for processing massive heterogeneous data and strengths in nonlinear pattern recognition, is emerging as a pivotal driver for pushing the knowledge boundaries in this field (15, 16). Current innovative applications of AI in neuroscience research primarily focus on BCI, neuroimaging analytics, and neurological disease prediction and diagnosis.

### 2.1 BCI: transitioning from movement control to human-AI integration

Deep learning-based EEG signal decoding techniques (e.g., CNN（Convolutional Neural Network: a deep learning model adept at processing grid-like data such as images or signals), EEGNet, ShallowNet, DeepCovNet) enable high-accuracy recognition of motor intent, accelerating the clinical translation of BCIs (17–19). Through implanted BCIs such as Neuralink's flexible electrode arrays, paralyzed patients can directly control robotic exoskeletons or cursor movements via neural activity patterns (20). Recent clinical reports demonstrate postoperative patients operating smart home systems and professional communication platforms within two weeks of implantation (21, 22). Non-invasive system integrates MEG/EEG with language models to achieve neural text decoding, enabling amyotrophic lateral sclerosis (ALS) patients to communicate via EEG-driven digital avatars (23, 24). Reinforcement learning algorithms (e.g., EPFL's inverse reinforcement learning: a type of machine learning where an agent learns to make decisions by receiving rewards or penalties) allow robots to dynamically correct movement trajectories by decoding error-related potentials (ErrPs) within 3–5 trials, realizing subject-specific obstacle avoidance and grasp control (25). AI facilitates BCI development by analyzing electroencephalographic (EEG) signals, enabling paralyzed patients to control external devices via neural commands (26, 27). Deep learning algorithms decode neural activity to operate robotic prosthetic limbs. Through high-throughput signal acquisition (e.g., 256-channel electrodes) (28) and adaptive decoding models [e.g., closed-loop decoder adaptation (CLDA)] (29), BCIs elucidate dynamic computational mechanisms underlying neural-behavioral mapping, establishing novel paradigms for neuroplasticity research.

### 2.2 Neuroimaging analytics: evolving from structural characterization to pathological prediction

AI overcomes the qualitative constraints of traditional imaging analysis, enabling quantitative integration of multimodal data. Nanobiosensors integrating surface-enhanced infrared absorption (SEIRA) spectroscopy with neural networks can noninvasively detect Parkinson's disease-associated misfolded protein oligomers in cerebrospinal fluid at single-molecule resolution (30). A research team at the Chinese Academy of Sciences demonstrated that combining OCTA (optical coherence tomography angiography) retinal scans with AI models enables Alzheimer's disease screening, which validated significant correlations between retinal microvascular density/fractal dimensions and cerebral amyloid-β deposition, offering a low-cost solution for primary care screening (31, 32). Transformer architectures(a deep learning model architecture using self-attention mechanisms, particularly effective for sequence data like time-series or text) decode fMRI temporal data to construct whole-brain connectome atlases, enabling precise localization of epileptogenic zones with submillimeter accuracy (<1 mm error) (33). Collectively, AI is fundamentally restructuring neuroimaging paradigms—from micropathological identification to macroscale functional prediction.

### 2.3 Neurological disease prediction and diagnosis: advancing from single biomarkers to multimodal integration

AI is shifting diagnostic windows earlier through dynamic bimodal monitoring of behavioral and physiological dimensions. Breakthroughs in early disease prediction have been achieved using AI—random forest models analyzing acoustic features (including Jitter and Shimmer) enable premotor diagnosis of Parkinson's disease before motor symptom onset (34). SMOTE (Synthetic Minority Over-sampling Technique: an algorithm that generates synthetic samples to address class imbalance in datasets) significantly enhances small-sample generalization capabilities (35). Domain-adaptive ridge regression models have been developed to predict UPDRS (Unified Parkinson's Disease Rating Scale) scores based on acoustic features of vowel/a/, achieving statistically significant error reduction through longitudinal patient data integration. This enables real-time tracking throughout therapeutic interventions (36). AI has revolutionized real-time diagnostic systems. Adapting real-time feedback mechanisms from educational contexts, BCI systems continuously monitor patient electroencephalographic (EEG) signals and generate diagnostic recommendations through integration with clinical knowledge bases. Combining abnormal slow-wave EEG power with APOE-*ε*4 genotypic data enables early warning of Alzheimer's disease risk (37). LSTM (Long Short-Term Memory: a type of recurrent neural network capable of learning long-term dependencies in sequence data) models decode spatiotemporal patterns of EEG spikes to forecast epileptic seizures pre-ictally, triggering responsive interventions (38). AI is systematically reconfiguring end-to-end clinical management of neurological disorders—from early latent-phase screening to real-time seizure prediction.

Three cross-cutting breakthroughs characterize AI's applications in neuroscience: algorithm-hardware co-evolution, accelerated clinical translation, and evolving ethical frameworks. For algorithm-hardware co-evolution, flexible electrodes (silk fibroin substrates) integrated with deep learning enable BCI miniaturization, reducing implantation trauma compared to conventional 256-channel arrays (39). Regarding clinical translation acceleration, China's《Brain-Computer Interface Industry Cultivation Plan (2025–2030)》prioritizes medical implementation; in August 2024, the NEO system became the country's first BCI product admitted to the Innovative Medical Devices Special Review Procedure, facilitating ambulatory recovery in spinal cord injury patients within 72 h post-implantation. For ethical framework development, Stanford scholars propose “Neurotechnology Principles” (reversibility, transparency, symbiosis), emphasizing safeguards against neural data exploitation and digital divides. Collectively, these advances propel neurotechnology toward high-efficiency, deep-integration, and strong-governance paradigms.

## 3 Multiscale integration of AI in biological research

AI is fundamentally restructuring life sciences research paradigms—enabling cross-scale data integration and mechanistic discovery from molecular-level precision manipulation to ecosystem-scale dynamic governance. Key breakthroughs are exemplified in gene editing and genomics, drug discovery, protein structure prediction, and ecological conservation.

### 3.1 Genome editing and genomics: advancing from precision enhancement to clinical translation

AI accelerates the design and optimization of genome-editing tools such as CRISPR (40). Deep learning models predict editing outcomes, enhancing efficiency in functional genomics and therapeutic development. Graph neural network (GNN)-based frameworks(a class of deep learning models designed to perform inference on data described by graphs)—including the CRISPR-ANT system developed by Feng Zhang's team—precisely forecast off-target effects while substantially improving editing efficiency (41–44). This technology achieved significantly higher hematopoietic stem cell correction rates than conventional methods in sickle cell disease gene therapy (45, 46). The DeepSEA framework precisely annotates the pathogenicity of non-coding mutations by analyzing millions of ENCODE epigenomic profiles, enhancing rare disease diagnostics (47). AI now serves as a pivotal translational accelerator, advancing genome editing from laboratory tool design toward precision clinical therapeutics.

### 3.2 Drug discovery: revolutionizing from virtual screening to end-to-end acceleration

AI significantly enhances efficiency in drug discovery by analyzing vast chemical and biological datasets to predict pharmacological properties—including activity, toxicity, and adverse effects—thereby accelerating compound screening and optimization (48–50). For instance, AI-driven drug discovery platforms identify potential anticancer agents within significantly reduced timeframes. Mirroring artificial neural network (ANN) architectures (computing systems inspired by biological neural networks) used for learning assessment in AI-enhanced educational settings, multilayer neural networks similarly optimize candidate molecule selection in pharmaceutical screening (13, 51). Generative AI models—such as Insilico Medicine's Chemistry42 platform integrated with reinforcement learning—designed the novel idiopathic pulmonary fibrosis inhibitor INS018_055 within seven days. INS018_055 represents a paradigm-shifting advance in AI-driven drug discovery, exemplified by its efficient identification of the TNIK kinase target, accelerated four-year trajectory from discovery to phase II trials, and rigorous multi-model therapeutic validation. This breakthrough underscores generative AI's transformative potential in addressing critical unmet medical needs (52). MetaTox multitask model, which integrates compound structure-metabolic pathway data, predicts hepatotoxicity with accuracy surpassing conventional animal testing, facilitating the replacement of toxicity assessments with “AI-organoid” systems (53–55). Leveraging multilayer perceptron architectures inspired by neural networks, Pfizer has developed a drug potency evaluation system that simulates molecule-target interactions through dynamic weight adaptation, reducing lead compound screening costs. The oncology candidate PF-07220060 (CDK4 inhibitor) was identified through virtual screening of 150,000 compounds, yielding 182 high-scoring molecules with a 38% experimental validation rate—surpassing the 8% industry average—accelerating its advancement to phase I clinical trials by 11 months (56). AI is fundamentally reconfiguring drug discovery paradigms from the molecular level, propelling end-to-end workflows toward intelligence-driven, highly efficient, and cost-effective transformation.

### 3.3 Protein structure prediction: advancing from single-chain folding to complex design

AI technologies, particularly deep learning models, have driven transformative advances in protein structure prediction (57, 58). While AlphaFold and RoseTTAFold represent breakthroughs, both face limitations in predicting disordered regions and dynamic complexes, highlighting the need for integrative experimental validation. For example, AlphaFold leverages deep learning algorithms to accurately determine three-dimensional protein structures, providing a pivotal tool for biological research and drug design (59, 60). AlphaFold 3 (2024) achieves groundbreaking accuracy in predicting protein-nucleic acid complexes, with a mere 1.2 Å prediction error for HIV capsid protein-viral RNA binding sites; its open-source model accelerates antivenom design, significantly improving toxin neutralization efficacy (61). AlphaFold 3 heralds the dawn of the “digital biology” era. Its significance extends beyond technical innovation to the democratization of science—empowering global research through freely accessible platforms and providing foundational tools for disease therapeutics, synthetic biology, and sustainable development. RoseTTAFold All-Atom (RFAA), developed by David Baker's team at the University of Washington, is a universal biomolecular modeling and design platform that transcends traditional protein structure prediction. It enables full-atom precision in modeling and designing complexes encompassing proteins, nucleic acids, small molecules, metal ions, and covalent modifications, establishing a new paradigm of “all-atom computational biology” (62). RoseTTAFold All-Atom enables precise calculation of antibody-antigen binding free energy (63). Moderna leveraged this capability to optimize mRNA vaccine carrier proteins, achieving significantly enhanced *in vivo* expression levels (64). Collectively, these advances signify AI's pivotal role in transitioning protein research from static structural resolution toward dynamic molecular interaction prediction and functional engineering.

### 3.4 Ecological conservation: transitioning from monitoring to intervention decision-making

AI analyzes ecological data to monitor species populations, predict environmental changes, and formulate conservation strategies (65). Acoustic recognition networks—such as the BirdNET system—process 100,000 h of field recordings to track population dynamics of critically endangered crested ibises (Nipponia nippon), substantially improving the efficiency of breeding habitat protection planning (66–68). Google Earth Engine (GEE) is an incredible coding interface for cloud processing of satellite imagery and data. GEE integrates spatiotemporal Transformer models to predict illegal logging hotspots in the Amazon rainforest using satellite imagery, enabling coordinated drone surveillance in real-time (69). Collectively, these applications empower ecological conservation to shift from passive monitoring toward intelligent decision-making, driving systemic enhancement of quantifiable ecological benefits.

AI is fundamentally reconfiguring the foundational logic of life sciences. At the microscopic mechanistic level, AlphaFold's revelation of the “sequence-structure-function” paradigm accelerates the transition from empirical observation to computational prediction. In research methodology innovation, generative AI transforms drug discovery from serendipity-driven screening to target-oriented design, achieving quantifiable cost reduction. For macro-ecological governance, spatiotemporal AI models enable vulnerability quantification across ecological networks, advancing biodiversity conservation within carbon neutrality framework. Collectively, AI drives a holistic paradigm shift—spanning microscopic deconstruction, meso-scale development, and macro-system governance—across the life sciences spectrum.

## 4 AI-driven transformation in healthcare: evolving from diagnostic assistance to precision intervention

Medicine represents one of the most extensively adopted domains for AI applications, with particularly prominent implementations in disease diagnosis, treatment, and health management (70). Medical AI is transcending traditional healthcare boundaries, reconfiguring end-to-end workflows spanning screening, diagnosis, treatment, and management.

### 4.1 Disease diagnostics: advancing from imaging analysis to multimodal integration

AI has achieved significant progress in medical imaging analysis (71, 72). For instance, FDA-approved AI-based SaMD tools such as IDx-DR for diabetic retinopathy demonstrate the clinical viability of AI in diagnostic imaging. Intelligent diagnostic imaging systems are undergoing substantial upgrades—the 3D-Transformer-based multimodal system for gastric cancer early screening. By integrating contrast-enhanced computed tomography (CE-CT) imaging with serum pepsinogen data, it significantly improves the detection rate of early gastric cancer (EGC) (73, 74). The Dr. Wise™ system (Deepwise) identifies microcalcification clusters in mammograms and integrates this imaging data with BRCA gene mutation data to construct individualized risk profiles. This approach significantly enhances the accuracy of early warnings for high-risk populations (75). Real-time intraoperative evaluation paradigm transformation. By adapting instant feedback mechanisms, originally conceptualized in educational settings, AI-powered intraoperative decision-support systems can complete frozen section analysis of tumor margins within 20 s. This advance significantly enhances the sensitivity for detecting positive margins and substantially reduces surgical duration. AI is propelling a fundamental evolution in disease diagnosis, shifting the paradigm from static image interpretation toward multimodal, real-time integrated decision-making. This transformation enables the establishment of a seamless clinical pathway encompassing pre-diagnostic risk stratification, intraoperative intervention, and post-treatment efficacy assessment—forming a comprehensive diagnostic-therapeutic loop. Despite high accuracy, AI diagnostic systems may exhibit bias in underrepresented populations, necessitating robust fairness audits.

### 4.2 Personalized therapeutics: from genetically informed stratification to adaptive optimization

AI technologies enable personalized therapeutic strategies by integrating patient genomic data, clinical records, and lifestyle factors. This approach enhances treatment efficacy while minimizing adverse effects. For instance, AI systems can recommend the most effective anticancer drugs based on an individual's specific genetic mutations. Analogous to the weighting methodology employed in learning quality assessment systems (e.g., the Analytic Hierarchy Process, AHP: a structured technique for organizing and analyzing complex decisions), AI assigns clinical significance weights to distinct genetic variants. This optimizes disease risk prediction models through data-driven prioritization (76, 77). AI system employs the AHP to assign clinical weights to driver mutations (e.g., EGFR, ALK), generating drug priority scores. This approach has significantly extended median overall survival in lung cancer patients (78, 79). DeepDR model integrates single-cell sequencing data with drug response profiles, anticipating osimertinib resistance four months prior to clinical manifestation. This enables timely therapeutic switching to brigatinib regimens (80, 81). The reinforcement learning simulates treatment-related toxicities, dynamically adjusting radiation doses for nasopharyngeal carcinoma patients. This strategy has significantly reduced parotid gland complication rates (82). AI is advancing personalized therapeutics beyond static genomic analysis toward dynamic optimization of living systems. This evolution establishes an end-to-end precision care loop encompassing risk prognostication, therapeutic decision-making, and real-time intervention.

### 4.3 Health management: from early risk alerting to proactive intervention

AI technology is finding increasingly extensive application in health management. By analyzing health data collected from wearable devices, AI can predict patients' health risks and deliver personalized health recommendations. For instance, AI algorithms can analyze data such as heart rate and blood pressure to predict an individual's risk of heart attack (83). Photoplethysmography- electrocardiography (PPG-ECG) fusion algorithm continuously monitors ST-segment deviations. It automatically triggers alerts to emergency response centres, significantly reducing myocardial infarction rescue response times (84). The leveraging federated learning analyzes dynamic glucose profiles alongside dietary records to generate personalized carbohydrate quantification advice. This approach has led to significant reductions in glycated hemoglobin levels among type II diabetes patients (85, 86). AI system utilizes voiceprint emotion recognition (analyzing Jitter/Shimmer features) to recommend cognitive behavioral therapy modules for individuals with depression, resulting in significantly improved (87). Collectively, AI is propelling health management beyond discrete risk warnings towards closed-loop proactive intervention systems, enabling continuous vital sign monitoring and personalized health optimization.

### 4.4 Robotic surgery: from precise manipulation to autonomous decision-making

AI-driven robotic surgical systems enable enhanced precision and minimally invasive procedures, reducing complications and accelerating patient recovery. For example, the flexible robotic arm with intraoperative AI vision module dynamically identifies vascular anomalies, significantly reducing blood loss during cholecystectomies (88). The AI system integrates DSA and MRI data for stereotactic procedures, achieving targeting errors of <0.3 mm and substantially improving hematoma evacuation efficiency in intracerebral hemorrhage cases (89). The AI system incorporates a reinforcement learning-based collision-avoidance module, which could autonomously navigate around critical neural bundles (e.g., preserving cavernous nerves during prostatectomy) (90). Collectively, AI is propelling surgical robotics beyond enhanced instrumentation towards quasi-autonomous agents with multimodal perception-decision-execution closed loops, thereby systematically redefining surgical safety margins and delivering quantifiable clinical benefits.

## 5 Cross-domain integration: the leap of the intelligent paradigm from education to healthcare

The core logic of AI—data-driven processing, personalization, and real-time feedback—reveals its universal applicability across domains, driving transformation from intelligent classrooms in education to AI-empowered medical research.

### 5.1 AI in medical education: from virtual simulation to clinical competency

Virtual laboratory platforms in biological education (e.g., Labster) enable students to simulate gene editing or drug synthesis processes through AI, reducing experimental costs while enhancing safety (91). Labster's virtual labs employ gamified interfaces that allow molecular-level manipulation of gene editing (e.g., simulating CRISPR off-target effects) and drug synthesis (e.g., virtual screening for SARS-CoV-2 inhibitors), achieving significant cost reductions and near-elimination of safety risks (92–94). AI system integrates force-feedback robotic arms to enable hands-on training assessments for endodontic procedures. The system provides real-time correction of student operational errors with short response latency, resulting in a significant improvement in skill assessment pass rates (95). The Virtual-Reality/Real-Printing platform integrates laparoscopic virtual simulation with pathological organ 3D printing. Within task-based modules, students complete comprehensive training spanning from imaging diagnosis to surgical planning, resulting in a significantly higher clinical thinking competency rate (96, 97). These systems share a “learner-centered” design core with smart classrooms. For instance, virtual simulation platform enables unlimited student repetition of high-risk procedures (e.g., cardiac catheterization) (98). This philosophy aligns with the “student-centered” instructional design of smart classrooms, emphasizing autonomous inquiry and real-time feedback (99, 100). Virtual laboratories are evolving from cost-saving tools into engines for clinical competency transformation. Propelled by immersive end-to-end training and intelligent closed-loop feedback systems, they are driving a paradigm shift in medical education towards autonomous exploration and precision assessment. These medical training applications illustrate how AI-driven simulation enhances clinical readiness, mirroring the personalized and feedback-driven logic of AI in healthcare.

### 5.2 Intelligent early-warning system: the dimensional upgrade of response from learning intervention to life rescue

Similar to early-warning mechanisms in learning quality assessment (e.g., threshold-triggered interventions) (101), AI systems in ICUs continuously monitor patient vital signs, generating real-time alerts for risks such as sepsis or cardiac arrest to secure critical intervention windows (102, 103). For instance, the multimodal model integrating dynamic indicators—including core temperature fluctuation trends and procalcitonin slope rates—significantly enhancing early-warning sensitivity and yielding alerts several hours earlier than conventional protocols (104). Similarly, the system identifies micro-variations in T-wave alternans (TWA) through ECG morphology analysis, initiating autonomous defibrillator pre-charging and substantially accelerating resuscitation response (105, 106). AI is thus transforming early-warning systems from learning aids into closed-loop command centers for life preservation. By compressing risk identification and response to millisecond timescales, it systematically redraws critical windows and survival probability curves in emergency medicine.

## 6 Challenges and future directions: building a trinity mechanism for tackling tough challenges of “technological breakthrough - ethical governance - talent recreation”

Emerging challenges include the use of synthetic data to mitigate data scarcity, model drift in longitudinal deployments, and the need for alignment with regulatory frameworks such as the FDA SaMD guidelines, EU AI Act, and China's Generative AI Measures. AI demonstrates substantial potential across neuroscience, biology, and medicine. However, its deep integration faces multifaceted challenges demanding systematic solutions. Regarding data barriers and privacy-security concerns, AI's heavy reliance on massive datasets makes patient privacy protection and data security paramount challenges. While open datasets (e.g., the US National Health and Nutrition Examination Survey, NHANES) offer accessibility, they carry inherent risks of misuse and can propagate misleading research (107). A critical challenge lies in the “black-box” nature and trust crisis of AI models. Particularly for deep learning models, their opaque decision-making processes hinder physician trust and undermine clinical adoption. The key imperative is enhancing model interpretability and ensuring robust generalization across diverse populations (e.g., patients in high-altitude regions) to mitigate misdiagnosis risks stemming from overfitting (108). A globally unified ethical framework for AI remains absent. In high-risk applications—such as surgical robotics—efficiency gains demand rigorous balancing against critical patient safety safeguards. Current regulatory systems struggle to keep pace with the accelerated evolution of these technologies (109, 110). Effective implementation of AI in biomedicine critically hinges on deep synergy among computer science, biology, and clinical medicine. However, cross-disciplinary collaboration mechanisms remain persistently underdeveloped (111). Unlocking the full potential of AI in life sciences and healthcare—and establishing a trustworthy new paradigm for intelligent medicine—requires addressing four critical imperatives: dismantling data silos, demystifying black-box models, forging ethical consensus, and bridging cross-disciplinary divides.

To systematically address these challenges, future initiatives must prioritize a tripartite synergistic framework integrating technological innovation, ethical governance, and workforce transformation. Develop interpretable AI (XAI) tools (methods that aim to make AI decision-making processes understandable to humans) to enhance model transparency and decision traceability, thereby strengthening clinical trust and adoption (112). Establish secure data-sharing ecosystems leveraging privacy-preserving techniques—such as federated learning (a machine learning approach where the algorithm is trained across multiple decentralized devices holding local data samples) and blockchain (a decentralized, distributed ledger technology)—to enable cross-institutional collaboration and data value extraction while rigorously safeguarding data privacy and security (113). Establish algorithmic accountability mechanisms incorporating human expert oversight (e.g., physician validation of AI recommendations) to ensure reliable, equitable, and auditable decision-making. Regulatory frameworks must integrate sustainability perspectives, proactively addressing societal impacts of deployment to mitigate risks of exacerbating healthcare disparities. Advance interdisciplinary education and practice through designing and implementing frontier curricula and research programs integrating AI, neuroscience, biology, and clinical medicine. This will systematically cultivate hybrid professionals with both technical mastery and domain expertise (114). As AI technologies undergo ongoing breakthroughs alongside advancements in this synergistic framework, their applications across neuroscience, biology, and medicine will expand in scope and depth. Through the convergent action of technological innovation, ethical governance, and workforce transformation, AI will overcome implementation bottlenecks for deep deployment in life and health sciences—restructuring medical paradigms towards an efficient, equitable, and sustainable revolutionary future. Global regulatory efforts such as the EU AI Act (2024) and WHO guidelines on AI in health provide foundational principles for accountability, transparency, and human oversight, yet harmonization remains a challenge.

## 7 Conclusions and perspectives

AI profoundly reshapes the fundamental principles of neuroscience, biology, and medicine, driving life sciences from experience-driven to “Data-Algorithm Dual Helix” paradigms. This study systematically demonstrates how AI overcomes bottlenecks in scale and efficiency inherent to traditional methods by: efficiently parsing massive heterogeneous data (e.g., enabling molecular-level detection of misfolded proteins in Parkinson's disease through multimodal fusion of neuroimaging data); deeply recognizing complex biological patterns (e.g., protein structure prediction); and responding to real-time dynamic systems (e.g., surgical robotics control). Concurrently, the personalized assessment logic prevalent in education (e.g., learning analytics systems) forms technical analogies with precision medical diagnostics and biological virtual experiment platforms (e.g., Labster), collectively validating the universal applicability of the “Data-Driven/Real-Time Feedback/Closed-Loop Optimization” paradigm. However, data-privacy barriers, algorithmic black-box effects, and collaborative fragmentation across disciplines remain core obstacles to intelligent implementation. Urgent needs include: building explainable AI tools to enhance clinical decision-making transparency, establishing secure data-sharing ecosystems via federated learning coupled with blockchain, and implementing interdisciplinary curricula to cultivate dual-qualified talent proficient in both technology and domain expertise. Looking forward, AI will drive deep convergence among neuroscience, biology, and medicine. Foundational models will evolve into “super brains”, optimizing brain injury repair strategies by integrating neuroplasticity mechanisms and predicting chronic disease risks using real-time metabolic data. Cross-disciplinary integration will catalyze innovations such as protein structure prediction-guided drug design for neurological disorders and BCI applications for personalized rehabilitation therapy. This convergence will redraw the cognitive boundaries of life sciences, ultimately enabling a paradigm shift from disease treatment to comprehensive intelligent life guardianship—encompassing gene-editing cures, active health management, and intelligent ecological governance. This transition aims to universalize medical resource access and ensure sustainable development. This AI-driven paradigm revolution is transforming life sciences from fragmented knowledge production to holistic intelligent guardianship, ushering humanity into a new era of equitable health and ecological sustainability.
